# Synthesis, Molecular Modelling and Biological Evaluation of Novel Heterodimeric, Multiple Ligands Targeting Cholinesterases and Amyloid Beta

**DOI:** 10.3390/molecules21040410

**Published:** 2016-03-26

**Authors:** Michalina Hebda, Marek Bajda, Anna Więckowska, Natalia Szałaj, Anna Pasieka, Dawid Panek, Justyna Godyń, Tomasz Wichur, Damijan Knez, Stanislav Gobec, Barbara Malawska

**Affiliations:** 1 Department of Physicochemical Drug Analysis, Faculty of Pharmacy, Jagiellonian University Medical College, Kraków 30-688, Poland; hebdamichalina@gmail.com (M.H.); marek.bajda@uj.edu.pl (M.B.); anna.wieckowska@uj.edu.pl (A.W.); natalia.szalaj@uj.edu.pl (N.S.); anna.pasieka@uj.edu.pl (A.P.); dawid.panek@uj.edu.pl (D.P.); justyna.godyn@uj.edu.pl (J.G.); tomasz.wichur@doctoral.uj.edu.pl (T.W.); 2 Department of Pharmaceutical Chemistry, Faculty of Pharmacy, University of Ljubljana, Ljubljana 1000, Slovenia; damijan.knez@ffa.uni-lj.si (D.K.); Stanislav.Gobec@ffa.uni-lj.si (S.G.)

**Keywords:** cholinesterase inhibitors, molecular modelling, β-amyloid aggregation inhibitors, Alzheimer’s disease, multi-target-directed ligands (MTDL), PAMPA-BBB assay

## Abstract

Cholinesterases and amyloid beta are one of the major biological targets in the search for a new and efficacious treatment of Alzheimer’s disease. The study describes synthesis and pharmacological evaluation of new compounds designed as dual binding site acetylcholinesterase inhibitors. Among the synthesized compounds, two deserve special attention—compounds **42** and **13**. The former is a saccharin derivative and the most potent and selective acetylcholinesterase inhibitor (*Ee*AChE IC_50_ = 70 nM). Isoindoline-1,3-dione derivative **13** displays balanced inhibitory potency against acetyl- and butyrylcholinesterase (BuChE) (*Ee*AChE IC_50_ = 0.76 μM, *Eq*BuChE IC_50_ = 0.618 μM), and it inhibits amyloid beta aggregation (35.8% at 10 μM). Kinetic studies show that the developed compounds act as mixed or non-competitive acetylcholinesterase inhibitors. According to molecular modelling studies, they are able to interact with both catalytic and peripheral active sites of the acetylcholinesterase. Their ability to cross the blood-brain barrier (BBB) was confirmed *in vitro* in the parallel artificial membrane permeability BBB assay. These compounds can be used as a solid starting point for further development of novel multifunctional ligands as potential anti-Alzheimer’s agents.

## 1. Introduction

Alzheimer’s disease (AD) is a fatal neurodegenerative disorder and the most frequent cause of dementia. World Health Organization estimates the global prevalence of the disease at 36 million [1] and annual costs at $604 billion [2]. These figures are expected to rise, yet we have no new drugs to ease the burden of the disease. Currently, only four drugs are used for the treatment of AD, the last of them being approved a decade ago. Also, an alarmingly low number of drug candidates are undergoing clinical trials, with only a dozen compounds in Phase III [3,4]. These facts highlight the necessity of new effective therapeutic agents for AD.

The etiology of AD is not entirely understood due to the heterogeneity of the disease where ageing, genetic and environmental risk factors play a very important role [5]. Two pathological changes in AD are senile plaques—deposits of amyloid beta peptide (Aβ) and neurofibrillary tangles (NFTs)—and aggregates of hyperphosphorylated tau protein [6]. These lesions accumulate in the affected brains and damage mainly the cholinergic neurons [7,8]. Severe loss of cholinergic neurons in the basal forebrain reduces the cholinergic activity in the cerebral cortex and hippocampus, and impairs memory and cognitive functions [9].

Restoration of memory impairments can be achieved by enhancing cholinergic neurotransmission through cholinesterase inhibition. Cholinesterases are enzymes responsible for acetylcholine (ACh) hydrolysis and thus termination of signal transmission. The first enzyme, acetylcholinesterase (AChE), hydrolyzes the majority (80%) of ACh, and the second, butyrylcholinesterase (BuChE), accounts for the remaining cholinesterase activity [10]. It was found that apart from its hydrolytic function, AChE is implicated in non-cholinergic functions such as amyloid deposition, cell adhesion and neurite outgrowth [11,12]. It is assumed that these functions of AChE are connected with its peripheral anionic site (PAS) [13]. The peripheral anionic site together with a catalytic anionic site (CAS) are the main binding sites of the enzyme [14].

Currently in AD therapy, there are either selective AChE inhibitors (donepezil, galantamine) or nonselective cholinesterase inhibitors (rivastigmine). Additionally to primary anti-cholinesterase activity, these drugs exhibit neuroprotective properties against Aβ toxicity, ischemia and glutamate excitotoxicity [15,16]. Senile plaques and neurofibrillary tangles are not the only culprits that exacerbate cholinergic neurotransmission. The pathogenesis of AD is far more complex, and other mechanisms—namely inflammation [17], oxidative stress [18], and immune suppression [19]—are also involved.

Taking into the consideration the wide range of factors involved in the onset and progress of AD, it is reasonable to apply the multiple ligand approach to discover new efficacious drugs for AD [20]. Multi-target-directed ligands (MTDL) are compounds which act on several biological targets simultaneously and possess noticeable advantages over single-target-directed ligands. With these drugs, the risk of drug-drug interactions, poor patient compliance and pharmacokinetic differences between the individual drugs can be avoided. Also, MTDL can be more efficacious and less vulnerable to resistance [21]. In recent years, many multifunctional compounds, which act on AD-relevant targets have been discovered and reviewed [22,23,24,25,26,27]. Among these, cholinesterase inhibitors with additional biological properties, such as Aβ-aggregation inhibition [28,29,30], monoamine oxidase inhibition [31,32,33], serotonergic activity [34,35] or neuroprotective properties [36], still represent the mainstay of research.

These studies represent a continuation of a project focused on the development of the MTDL as potential anti-AD agents [37,38,39,40,41]. Herein, we describe the design, molecular modelling and synthesis of a new series of heterodimeric compounds. Their biological activity was evaluated on three potential targets: acetylcholinesterase (AChE), butyrylcholinesterase (BuChE) and Aβ_1__–42_ aggregation. Finally, their blood–brain barrier (BBB) permeability was assessed using the parallel artificial membrane permeation assay (PAMPA—BBB).

## 2. Results and Discussion

### 2.1. Design

**Figure 1:**
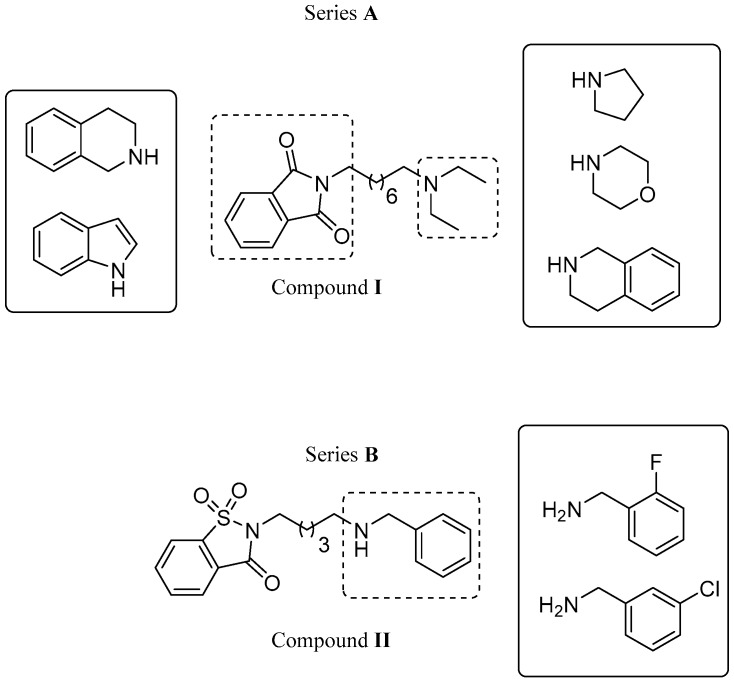
The general structures of the designed compounds.

Two series of compounds were designed as dual binding site acetylcholinesterase inhibitors based on our previous research. Compound **I**, a selective and moderate *Ee*AChE inhibitor (IC_50_ = 0.90 µM), and a weak self-induced Aβ_12–23_ aggregation inhibitor (30.10% at 50 µM) were used as a starting point for series A [37]. Diethylamine moiety of compound **I** was replaced with cyclic analogues (pyrrolidine, morpholine and tetrahydroisoquinoline). Phthalimide on the left-hand side of **I** was replaced with tetrahydroisoquinoline and an indole group. Both fragments were connected by alkyl linkers of various lengths (Figure 1).

Series **B** was designed on the basis of a potent and selective saccharin-benzylamine based AChE inhibitor (compound **II**, *Ee*AChE IC_50_ = 36 nM and 22.19% of inhibition of self-induced Aβ_1–42_ aggregation at 10 μM) [40]. The chemical space was probed by modifications of the benzylamine fragment (Figure 1).

### 2.2. Chemistry

**Scheme 1:**
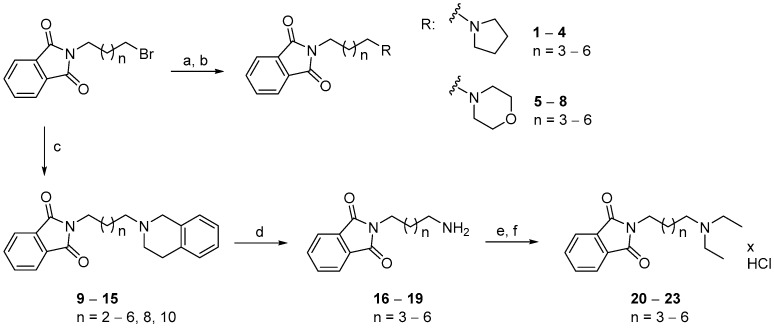
Synthesis of the isoindoline-1,3-dione derivatives **1**–**15** and tetrahydroisoquinoline derivatives **20**–**23**. *Reagents and conditions*: (**a**) pyrrolidine or morpholine, K_2_CO_3_, MeCN, reflux, 20 h; (**b**) HCl in 2-propanol; (**c**) tetrahydroisoquinoline, K_2_CO_3_, MeCN, reflux, 24 h; (**d**) *i*: NH_2_NH_2_, EtOH, reflux, 2 h; *ii*: 0 °C, 36% HCl, reflux, 2 h (**e**) bromoethane, K_2_CO_3_, MeCN, rt, 48 h; (**f**) HCl in 2-propanol.

The synthesis of the designed isoindoline-1,3-dione derivatives was accomplished as shown in Scheme 1. 2-(ω-Bromoalkyl)isoindoline-1,3-diones were used as alkylating agents in a reaction of nucleophilic substitution with secondary amines (pyrrolidine, morpholine and tetrahydroisoquinoline). The reactions were carried out in acetonitrile (MeCN) in the presence of potassium carbonate for 24 h, under reflux. Following purification by silica gel column chromatography, the final 2-(ω-(*N*-amino)alkyl)isoindoline-1,3-dione derivatives **1**–**15** were isolated and converted into their hydrochloride salts. Compounds **10**–**13** were further hydrolyzed in reaction with ethanolic solution of hydrazine under reflux. After 2 h, the reaction mixture was cooled down to 0 °C and 36% HCl was added and mixed for the next 2 h, under reflux. Amines **16**–**19** were isolated and alkylated with bromoethane in the presence of potassium carbonate. The reactions were carried out in acetonitrile for 48 h, at room temperature. Following purification by silica gel column chromatography, the final ω-(3,4-dihydroisoquinolin-2(1*H*)-yl)-*N*,*N*-diethylalkyl-1-amine derivatives **20**–**23** were isolated and subsequently converted into hydrochloride salts.

**Scheme 2:**
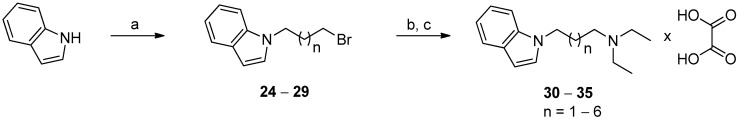
Synthesis of the indole derivatives **30**–**35**. *Reagents and conditions*: (**a**) α,ω-dibromoalkane, (CH_3_)_3__3_CO^−^K^+^, THF, 0 °C, 3 h (**b**) diethylamine, K_2_CO_3_, MeCN, reflux 24 h; (**c**) Oxalic acid in acetone.

A subseries of indole derivatives was synthesized according to the reaction protocol depicted in Scheme 2. In the first step, indole was alkylated with the appropriate α,ω-dibromoalkane in the presence of potassium *tert*-butoxide ((CH_3_)_3_CO^−^K^+^). The reactions were carried out in anhydrous tetrahydrofuran (THF) at 0 °C for 3 h. In the next step, compounds **24**–**29** were used as alkylating agents in a reaction with diethylamine. The reactions were carried out in acetonitrile, in the presence of potassium carbonate, stirring for 24 h under reflux. After purification by silica gel column chromatography, the final *N*,*N*-diethyl-ω-(1*H*-indol-1-yl)alkyl-1-amine derivatives **30**–**35** were isolated and converted into oxalates.

**Scheme 3:**
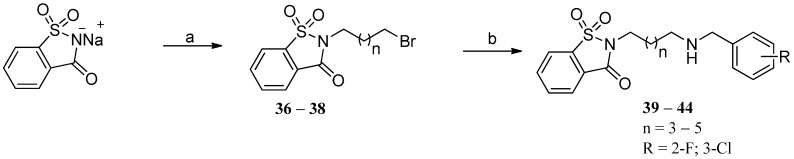
Synthesis of the saccharin derivatives **39**–**44**. *Reagents and conditions*: (**a**) α,ω-dibromoalkane, DMF, reflux, 24 h; (**b**) 2-fluorobenzylamine or 3-chlorobenzylamine, DMSO, 60 °C, 3.5 h.

Series **B** of benzo[*d*]isothiazol-3(2*H*)-one 1,1-dioxide derivatives was prepared according to the pathway described in Scheme 3. In the first step, alkylation of a saccharin sodium salt with the appropriate α,ω-dibromoalkane gave intermediate compounds **36**–**38**. Reactions were carried out in *N*,*N*-dimethylformamide (DMF) for 24 h under reflux, followed by purification with flash column chromatography. Subsequently, compounds **36**–**38** were used as alkylating agents in reaction of nucleophilic substitution with 2-fluorobenzylamine or 3-chlorobenzylamine. The reactions were carried out in dimethyl sulfoxide (DMSO) for 3.5 h at 60 °C, followed by silica gel flash chromatography purification, which afforded the expected 2-(ω-(*N*-benzylamino)alkyl)benzo[*d*]isothiazol-3(2*H*)-one 1,1-dioxides **39**–**44**.

### 2.3. Biological Evaluation

#### 2.3.1. Cholinesterase Inhibitory Potency

Ellman’s method [42] was used to determine the activity of the synthesized compounds against AChE from electric eel (*Electrophorus electricus*, *Ee*AChE) and BuChE from equine serum (*Eq*BuChE). The results of the assays are presented in Table 1. Tacrine, donepezil as well as compounds **I** and **II** were included as references.

In the series **A**, moderate *Ee*AChE inhibitors with IC_50_ values ranging from 0.276 µM to 22.185 µM were identified. When assessing the impact of the amine moiety on *Ee*AChE inhibitory potency, we found that the replacement of diethylamine (compound **I**) by pyrrolidine (compounds **1**–**4**) or tetrahydroisoquinoline (compounds **11**–**13**) increased the potency on *Ee*AChE. Only in the case of morpholine, we observed a significant decrease in potency (compounds **5**–**8**). The most potent was compound **3** (*Ee*AChE IC_50_ = 0.276 µM) with the pyrrolidine moiety. Regarding the length of the linker, the most potent were the compounds with six to eight carbon atom linkers. The potency decreased with shortening and further elongation of the tether. Compounds **14** and **15** with the longest linkers (10 and 12 carbon atoms) were not active. Modifications of the heteroaromatic fragment also led to reduced potencies of the tested compounds. These compounds (**20**–**23**, **30**–**35**) were less potent in comparison with parent compound **I** and inhibited the enzyme in high micromolar range.

On the basis of the Ellman’s assay for series **B**, we found that this series inhibited *Ee*AChE in the low micromolar to nanomolar range. Compounds with a five carbon atom linker: **39** (2-fluoro derivative) and **42** (3-chloro derivative) were the most potent *Ee*AChE inhibitors with IC_50_ values of 150 and 70 nM, respectively. Comparing these compounds with their unsubstituted analogue (compound **II**, *Ee*AChE IC_50_ = 36 nM), it was established that neither the introduction of a fluorine atom nor the introduction of a chlorine atom led to significant benefits in the terms of inhibitory potency. Compounds **39** and **42** were also the most potent and most selective *Ee*AChE inhibitors presented in this paper.

*Eq*BuChE inhibitory studies showed that the half of the tested compounds were weak *Eq*BuChE inhibitors with IC_50_ values ranging from 0.618 to 37.129 µM and the other half were selective *Ee*AChE inhibitors. The most potent were compounds with seven and eight carbon linkers while shortening or elongation of the linker resulted in the decrease of potency similar to series A. Compound **13**, bearing phthalimide and tetrahydroisoquinoline moieties, was the most potent *Eq*BuChE inhibitor (IC_50_ = 0.618 µM) and a moderate *Ee*AChE inhibitor (IC_50_ = 0.760 µM) from this series.

#### 2.3.2. Kinetic Studies of AChE Inhibition

**Figure 2:**
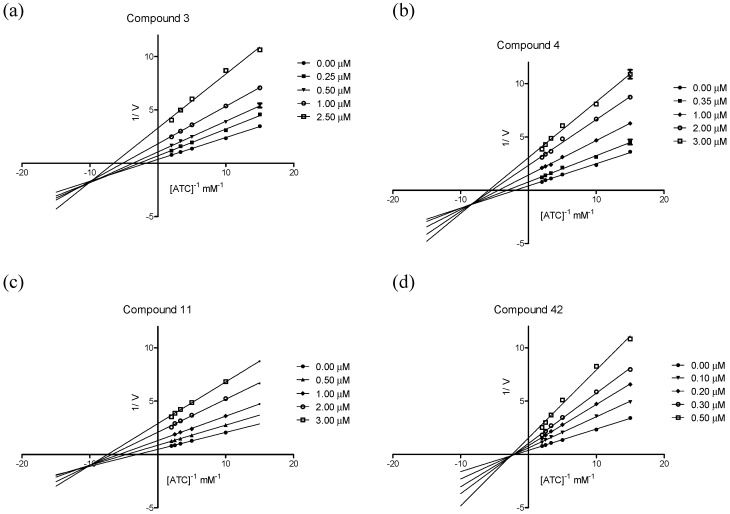
Lineweaver–Burk plots illustrating mixed type of *Ee*AChE inhibition by compounds **3** (**a**); **4** (**b**); **11** (**c**) and non-competitive type of *Ee*AChE inhibition by compound **42** (**d**). ATC = acetylthiocholine; *V* = initial velocity rate.

For kinetic studies, we have chosen compounds **3** and **4** with the pyrrolidine moiety and **11** with the tetrahydroisoquinoline moiety, as the most potent AChE inhibitors from series A. Compound **42** was chosen as the most potent AChE inhibitor from series B. Initial velocity dependence on different substrate concentration in the absence and presence of the tested compounds at six different concentrations was established using Ellman’s method. Analysis of Lineweaver–Burk double reciprocal plots showed that compounds **3**, **4** and **11** display mixed type of enzyme inhibition (partial competitive and pure non-competitive) as demonstrated by increased slopes (decreased Vmax) and decreased intercepts (lower Km) at increasing concentration of the inhibitor (Figure 2a–c). For the compound **42**, we observed increased slopes and preserved intercepts at increasing concentrations of the inhibitor, which characterize a linear non-competitive type of inhibition (Figure 2d). Both types of inhibition were further confirmed by Cornish–Bowden plots (S/*V* *vs.* [I]) (A. in Supplementary Materials) [43].

#### 2.3.3. Aβ_1–42_ Aggregation Inhibitory Potency

Mechanisms of Aβ aggregation remain unclear. Besides the self-induced assembly of Aβ, several other factors affect its aggregation, including metal ions [44], AChE [45], and oxidative stress [46]. Therefore, we evaluated our compounds using the most versatile and commonly used Aβ_1–42_ aggregation Thioflavin T assay [47]. Seven structurally diverse compounds were selected (one from each subseries) to test their ability to inhibit self-induced Aβ_1–42_ aggregation. The results of this assay showed that these derivatives are rather weak inhibitors of Aβ aggregation at 10 µM. Only compound **13** was found to be a moderate inhibitor with the 35.80% ± 5.39% inhibition of Aβ_1–42_ aggregation. Even though it displayed higher potency than donepezil in this assay, compound **13** was a less potent cholinesterase inhibitor than the reference drug, so the multitarget profile of this compound still needs to be optimized.

### 2.4. Molecular Modelling Studies

The structure of *Torpedo californica* AChE (*Tc*AChE) and the previously described fragment-based approach were used in molecular modelling studies [48]. Our biological assay was performed on *Electrophorus electricus* AChE. However, for docking *Tc*AChE of resolution 2.5 Å, obtained from Protein Data Bank (PDB code: 1EVE), was utilized. It contained donepezil molecule—reference ligand which was structurally similar to our compounds. Sequence alignment of *Tc*AChE and *Ee*AChE revealed a very high degree of identity (above 60%). Further analysis showed that there was only one residue difference in the active site. The Phe330 residue in *Torpedo californica* was replaced by Tyr in *Electric eel* enzyme [49]. This justified application of *Tc*AChE for the docking studies.

Designed compounds were docked into the active site of AChE to find possible interactions with the enzyme. The binding potency of novel inhibitors was assessed by the ChemScore function and the poses were inspected visually.

**Figure 3:**
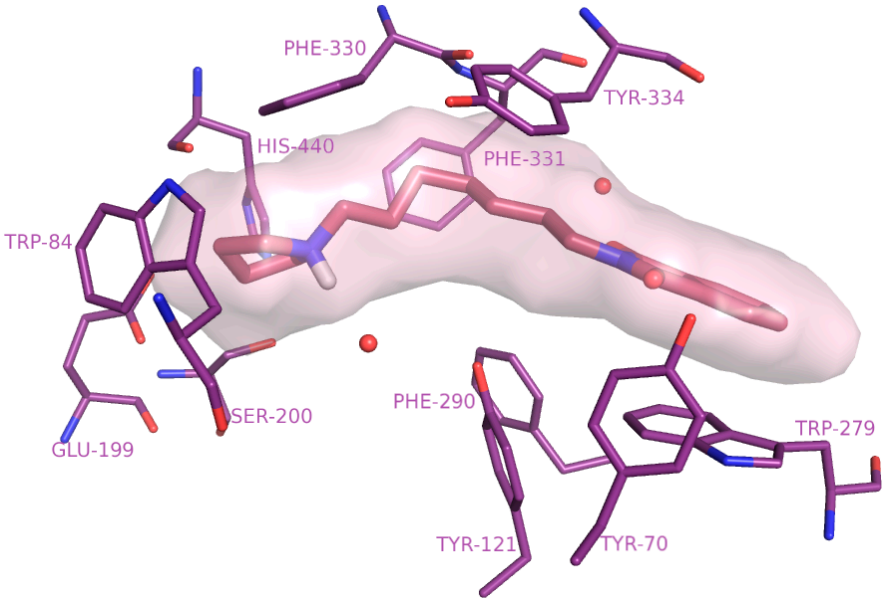
The binding mode of compound **3** (pink) within the active site of AChE.

The first subgroup of inhibitors (compounds **1**–**4**) were simple analogues of parent compound **I** and interacted with AChE in a similar way [37]. The binding mode for the most active inhibitor **3** from this subgroup is presented in Figure 3. The protonated amine group formed cation-π interactions with aromatic amino acids in the anionic subsite (Phe330 and Trp84) and a hydrogen bond network with the hydroxyl group of Tyr121 via a water molecule. The pyrrolidine, a cyclic analogue of diethylamine, provided stronger hydrophobic interactions with Trp84 and, therefore, derivatives **1**–**4** were stronger inhibitors of AChE in comparison with compound **I**. The phthalimide moiety of these inhibitors was engaged in interactions with aromatic amino acids in the PAS: π–π stacking with Trp279 and CH–π interactions with Tyr70. Moreover, both carbonyl groups formed hydrogen bonds: one with Tyr121 and the other with a water molecule. The optimal linker length was equal to 7–8 methylene groups. Such tether enabled the terminal fragments to interact with Trp84 and Trp279 in an optimal way. The aliphatic linker also formed hydrophobic interactions with aromatic residues (Phe290, Phe331 and Tyr334) half-way down the active site gorge.

The second subset (compounds **5**–**8**) had an enlarged heterocyclic ring—morpholine. Morpholine could provide an extra hydrogen bond between the oxygen atom and the hydroxyl group of Ser200 from the catalytic triad of AChE. However, it required a shift of heterocyclic ring towards the serine in comparison with pyrrolidine. This change led to reduced cation–π interactions, especially with Phe330 resulting in lower potency.

**Figure 4:**
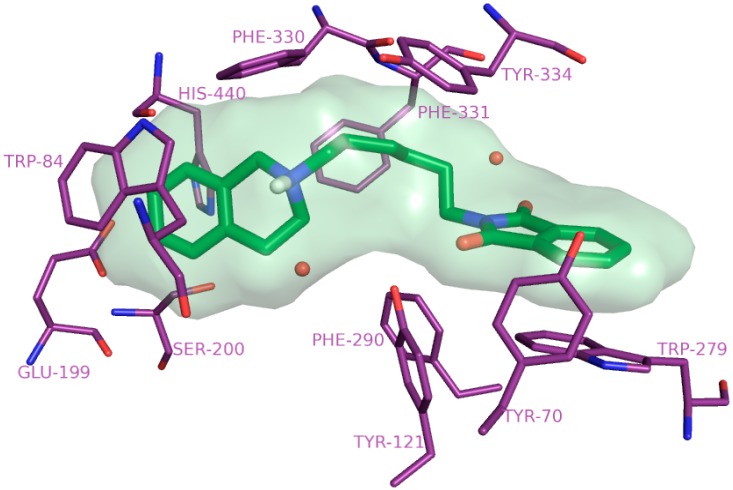
The binding mode of compound **11** (green) within the active site of AChE.

In the case of the third subgroup (derivatives **9**–**15**), tetrahydroisoquinoline was introduced instead of diethylamine. The most potent inhibitor **11** with tetrahydroisoquinoline ring docked into acetylcholinesterase is shown in Figure 4. This modification was beneficial because the combination of an aromatic ring and aliphatic amine gave π–π stacking with Trp84 as well as cation–π interaction with Phe330. The optimal linker was found to be a six-carbon atom aliphatic chain.

Further structural modifications of parent inhibitor **I** gave the next two subsets: the fourth (compounds **20**–**23**) and the fifth (derivatives **30**–**35**). In both cases, the phthalimide was replaced with heteroaromatic moiety: tetrahydroisoquinoline and indole, respectively. Tetrahydroisoquinoline provided π–π stacking as well as cation–π interaction with Trp279, and indole stronger π–π stacking. However, the lack of hydrogen bonds which were previously formed with two carbonyl groups of phthalimide reduced the potency.

**Figure 5:**
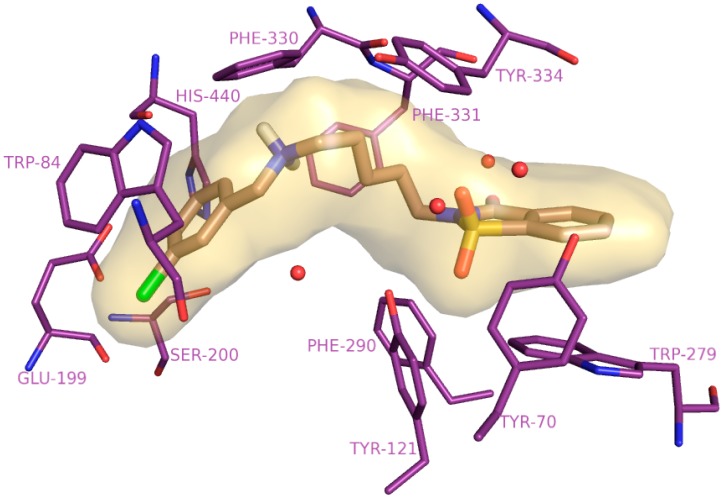
The binding mode of compound **42** (dark salmon) within the active site of AChE.

The last two subgroups were based on compound II [40]. The binding mode of the most potent inhibitor 42 is presented in Figure 5. The sixth subset [39,40,41] contained a fluorine atom while the seventh one [42,43,44] had a chlorine atom on the benzylamine fragment. Introduction of the fluorine atom at *ortho* position might provide a hydrogen bond with Ser200 while a chlorine atom at *meta* position a halogen bond with the carboxyl group of Glu199 or backbone of Gly441 upon small shift and/or rotation of benzyl substituent. However, the halogen substituted derivatives revealed the same binding mode as parent inhibitor II. The benzyl moiety was π–π stacked with Trp84 in the CAS. Orientation of this fragment remained the same as for parent compound II, and no beneficial interactions were observed with halogen atoms. The saccharin fragment was engaged in π–π stacking with Trp279 and CH–π interactions with Tyr70 in the PAS. The carbonyl group formed an H-bond with a water molecule while the oxygen atoms of sulfone formed H-bonds with Tyr121 and two other water molecules. The protonated amino group formed cation–π interactions with Phe330 and a hydrogen bond network with Tyr121 via a water molecule. The alkyl linker formed hydrophobic interactions with aromatic residues such as Phe290, Phe331, and Tyr334 located halfway down the active gorge.

Summing up, all subseries were able to interact simultaneously with both the catalytic and peripheral active sites of acetylcholinesterase. However, the quality of the predicted interactions varies substantially and may thus lead to the diverse range of activity. The dual binding mode is characteristic for donepezil as well as for previously described isoindoline-1,3-dione and benzo[*d*]isothiazol-3(2*H*)-one 1,1-dioxide derivatives.

### 2.5. Blood–Brain Barrier Permeability Assay

For anti-Alzheimer drug candidates, the ability to cross the blood–brain barrier and to enter the central nervous system (CNS) is crucial to achieve their pharmacological target and activity. Therefore, the blood–brain barrier permeability of the selected compounds **4**, **7**, **13**, **39** and **4**2 was assessed using the parallel artificial membrane permeability assay (PAMPA-BBB) [50]. We used seven commercial drugs as references that allowed us to establish the following ranges of permeability: log P_e_ > −4.5 for compounds with high permeability, log P_e_ ≤ −6.3 for compounds with low permeability and −6.3 < log P_e_ ≤ −4.5 for compounds with uncertain permeability (Table 2). According to the results summarized in Table 2, all off the tested compounds should be able to cross BBB and reach CNS.

## 3. Materials and Methods

### 3.1. Chemistry

#### 3.1.1. General Methods

^1^H-NMR spectra were recorded on Varian Mercury 300 (Varian, San Diego, CA, USA) at 300 MHz. The chemical shifts for ^1^H-NMR are referenced to residual solvent signals (^1^H, CDCl_3_ at 7.26 ppm, DMSO-*d*_6_ at 2.50 ppm). Mass spectra (MS) were obtained on an UPLC-MS/MS system consisting of a Waters ACQUITY^®^ UPLC^®^ (Waters Corporation, Milford, MA, USA) coupled to a Waters TQD mass spectrometer (electrospray ionization mode ESI-tandem quadrupole). Analytical thin layer chromatography (TLC) was done using aluminum sheets precoated with silica gel 60 F254 (Merck, Darmstadt, Germany). Column chromatography was performed on Merck silica gel 60 (63–200 μm) (Merck). For the TLC and column chromatography following solvent systems were used: S_1_ (*n*-hexane, ethyl acetate, trimethylamine (TEA); 5:5:1, *v*/*v*), S_2_ (petroleum ether, DCM; 8:2, *v*/*v*), S_3_ (DCM, MeOH; 9.5:0.5, *v*/*v*/*v*), S_4_ DCM, MeOH; 9:1, *v*/*v*), S_5_ (DCM, MeOH; 10:1, *v*/*v*), S_6_ (DCM, petroleum ether, MeOH, TEA; 5:3.5:1.5:3 drops, *v*/*v*/*v*/*v*). The purity of the final compounds was determined using an analytical RPLC-MS on Waters Acquity TQD using an Aquity UPLC BEH C18 column (1.7 μm, 2.1 × 100 mm) at 214 nm and 254 nm. CH_3_CN/H_2_O gradient with 0.1% HCOOH was used as the mobile phase at a flow rate of 0.3 mL/min. All the compounds showed purity above 95%, as determined by RPLC. Melting points were determined in open capillaries on an Electrothermal 9300 apparatus (Electrothermal, Staffordshire, UK) and are uncorrected. Elemental analyses were performed on Vario EL III Elemental analyser (Elementar Analysensysteme GmbH, Hanau, Germany). All the reagents were purchased from commercial suppliers and were used without further purification. Tetrahydrofuran (THF) and dichloromethane (DCM) were distilled under nitrogen immediately before use. The drying agent used for THF was sodium benzophenone ketyl, and for DCM, calcium hydride.

The following compounds: 2-(5-bromopentyl)isoindoline-1,3-dione [37], 2-(6-bromohexyl)isoindoline-1,3-dione [37], 2-(7-bromoheptyl)isoindoline-1,3-dione [37], 2-(8-bromooctyl)isoindoline-1,3-dione [37], 2-(10-bromodecyl)isoindoline-1,3-dione [51], 2-(12-bromododecyl)isoindoline-1,3-dione [51], 2-(4-(bromomethyl)benzyl)isoindoline-1,3-dione [52], 1-(3-bromopropyl)-1*H*-indole [53], 1-(4-bromobutyl)-1*H*-indole [53], 1-(5-bromopentyl)-1*H*-indole [53], 1-(6-bromohexyl)-1*H*-indole [54], 1-(7-bromoheptyl)-1*H*-indole [54], 1-(8-bromooctyl)-1*H*-indole [54], 2-(5-(pyrrolidin-1-yl)pentyl)isoindoline-1,3-dione (**1**) [55], 2-(5-morpholinopentyl)isoindoline-1,3-dione (**5**) [56], 2-(8-morpholinooctyl)isoindoline-1,3-dione (**8**) [57], 2-(4-(3,4-dihydroisoquinolin-2(1*H*)-yl)butyl)isoindoline-1,3-dione (**9**) [58], 2-(5-(3,4-dihydroisoquinolin-2(1*H*)-yl)pentyl)isoindoline-1,3-dione (**10**) [59], 2-(6-(3,4-dihydroisoquinolin-2(1*H*)-yl)hexyl)isoindoline-1,3-dione (**11**) [59], 2-(12-(3,4-Dihydroisoquinolin-2(1*H*)-yl)dodecyl)isoindoline-1,3-dione (**15**) [60], 5-(3,4-dihydroisoquinolin-2(1*H*)-yl)pentan-1-amine (**16**) [61], 6-(3,4-dihydroisoquinolin-2(1*H*)-yl)hexan-1-amine (**17**) [53], 7-(3,4-dihydroisoquinolin-2(1*H*)-yl)heptan-1-amine (**18**) [54], 8-(3,4-dihydroisoquinolin-2(1*H*)-yl)octan-1-amine (**19**) [54], 2-(5-bromopentyl)benzo[*d*]isothiazol-3(2*H*)-one 1,1-dioxide (**36**) [62], 2-(6-bromohexyl)benzo[*d*]isothiazol-3(2*H*)-one 1,1-dioxide (**37**) [62], 2-(7-bromoheptyl)benzo[*d*]isothiazol-3(2*H*)-one 1,1-dioxide (**38**) [59] have been reported previously.

#### 3.1.2. General Procedure for the Preparation of Hydrochloride Salts

The hydrochloride salts were prepared by dissolving the compounds in a minimum quantity of dichloromethane. The solution was then treated with 5 M solution of HCl in 2-propanol, evaporated under reduced pressure, washed with diethyl ether and dried. 

#### 3.1.3. General Procedure for the Synthesis of Compounds (**1**–**8**)

Procedure M1. A mixture of the appropriate 2-(bromoalkyl)isoindoline-1,3-dione (1 equiv) with amine (pyrrolidine or morpholine) (1.1 equiv) in the presence of K_2_CO_3_ (3 equiv) was stirred in acetonitrile under reflux for 20 h. Subsequently, the solvent was evaporated under reduced pressure, producing a residue which was further dissolved in 20 mL of water and extracted with DCM (3 × 30 mL). The organic layer was dried with anhydrous Na_2_SO_4_. The solvent was then evaporated and the residue was purified by silica gel column chromatography (S_3_) yielding a yellow oil. The final product was transformed into hydrochloride salt. The following compounds were obtained.

*2-(5-(Pyrrolidin-1-yl)pentyl)isoindoline-1,3-dione* (**1**) [55]. Procedure M1. Reaction of 2-(5-bromopentyl)isoindoline-1,3-dione [37] (0.5 g, 1.69 mmol) with pyrrolidine (0.13 g, 1.86 mmol) and K_2_CO_3_ (0.7 g, 5.1 mmol) in acetonitrile (25 mL), after 20 h, column chromatography gave oil product. Yield 0.35 g (73%). TLC (S_3_) *R_f_* = 0.13. MW 286.17. Formula: C_17_H_22_N_2_O_2_. MS: *m*/*z* 287.28 [M + H]^+^. ^1^H-NMR (300 MHz, CDCl_3_) δ ppm: 7.89–7.77 (m, 2H), 7.78–7.65 (m, 2H), 3.69 (t, *J* = 6.9 Hz, 2H), 3.00 (m, 4H), 2.06–1.88 (m, 4H), 1.81–1.65 (m, 4H), 1.48–1.21 (m, 4H). Hydrochloride salt: M.p. 190 °C. Elemental analyses (%) for C_17_H_22_N_2_O_2_·HCl Calc. C 63.25; N 8.63; H 7.18, found: C 62.73; N 8.54; H 7.27.

*2-(6-(Pyrrolidin-1-yl)hexyl)isoindoline-1,3-dione* (**2**). Procedure M1. Reaction of 2-(6-bromohexyl)isoindoline-1,3-dione [37] (0.65 g, 2.1 mmol) with pyrrolidine (0.16 g, 2.3 mmol) and K_2_CO_3_ (0.87 g, 6.28 mmol) in acetonitrile (25 mL), after 20 h, column chromatography gave oil product. Yield 0.44 g (70%). TLC (S_3_) *R_f_* = 0.15. MW 300.18. Formula: C_18_H_24_N_2_O_2_. MS: *m*/*z* 301.31 [M + H]^+^. ^1^H-NMR (300 MHz, CDCl_3_) δ ppm: 7.87–7.77 (m, 2H), 7.74–7.65 (m, 2H), 3.66 (t, *J* = 7.1 Hz, 2H), 2.59 (t, *J* = 6.7 Hz, 4H), 2.49 (t, *J* = 7.2 Hz, 2H), 1.87–1.76 (m, 4H), 1.73–1.50 (m, 4H), 1.41–1.31 (m, 4H). Hydrochloride salt: M.p. 151 °C. Elemental analyses (%) for C_18_H_24_N_2_O_2_·HCl Calc. C 64.18; N 8.32; H 7.48, found: C 64.07; N 8.13; H 7.73. 

*2-(7-(Pyrrolidin-1-yl)heptyl)isoindoline-1,3-dione* (**3**). Procedure M1. Reaction of 2-(7-bromoheptyl)isoindoline-1,3-dione [37] (0.648 g, 2 mmol) with pyrrolidine (0.156 g, 2.2 mmol) and K_2_CO_3_ (0.83 g, 6 mmol) in acetonitrile (25 mL), after 20 h, column chromatography gave oil product. Yield 0.49 g (78%). TLC (S_3_) *R_f_* = 0.24. MW 314.20. Formula: C_19_H_26_N_2_O_2_. MS: *m*/*z* 315.40 [M + H]^+^. ^1^H-NMR (300 MHz, CDCl_3_) δ ppm: 7.88–7.75 (m, 2H), 7.75–7.62 (m, 2H), 3.65 (t, *J* = 7.2 Hz, 2H), 2.57 (t, *J* = 7.6 Hz, 4H), 2.46 (t, *J* = 6.7 Hz, 2H), 1.86–1.73 (m, 4H), 1.71–1.47 (m, 4H), 1.39–1.23 (m, 6H). Hydrochloride salt: M.p. 115 °C. Elemental analyses (%) for C_19_H_26_N_2_O_2_·HCl Calc. C 65.04; N 7.98; H 7.76, found: C 65.24; N 7.76; H 7.88.

*2-(8-(Pyrrolidin-1-yl)octyl)isoindoline-1,3-dione* (**4**). Procedure M1. Reaction of 2-(8-bromooctyl)isoindoline-1,3-dione [37] (0.678 g, 2 mmol) with pyrrolidine (0.156 g, 2.2 mmol) and K_2_CO_3_ (0.83 g, 6 mmol) in acetonitrile (25 mL), after 20 h, column chromatography gave oil product. Yield 0.53 g (80%). TLC (S_3_) *R_f_* = 0.23. MW 328.22. Formula: C_20_H_28_N_2_O_2_. MS: *m*/*z* 329.43 [M + H]^+^. ^1^H-NMR (300 MHz, CDCl_3_) δ ppm: δ 7.88–7.75 (m, 2H), 7.75–7.62 (m, 2H), 3.65 (t, *J* = 7.2 Hz, 2H), 2.54 (t, *J* = 6.9 Hz, 4H), 2.44 (t, *J* = 7.8 Hz, 2H), 1.85–1.72 (m, 4H), 1.70 – 1.43 (m, 4H), 1.36 – 1.21 (m, 8H). Hydrochloride salt: M.p. 110 °C. Elemental analyses (%) for C_20_H_28_N_2_O_2_·HCl Calc. C 65.83; N 7.68; H 8.01, found: C 65.88; N 7.73; H 8.17.

*2-(5-Morpholinopentyl)isoindoline-1,3-dione* (**5**) [56]. Procedure M1. Reaction of 2-(5-bromopentyl)isoindoline-1,3-dione [37] (0.51 g, 1.72 mmol) with morpholine (0.17 g, 1.89 mmol) and K_2_CO_3_ (0.75 g, 5.15 mmol) in acetonitrile (25 mL), after 20 h, column chromatography gave oil product. Yield 0.51 g (89%). TLC (S_3_) *R_f_* = 0.41. MW 302.16 Formula: C_17_H_22_N_2_O_3_. MS: *m*/*z* 303.24 [M + H]^+^. ^1^H-NMR (300 MHz, CDCl_3_) δ ppm: 7.89–7.76 (m, 2H), 7.76–7.63 (m, 2H), 3.73–3.61 (m, 6H), 2.40 (t, *J* = 4.4 Hz, 4H), 2.30 (t, *J* = 7.5 Hz, 2H), 1.77–1.60 (m, 2H), 1.60–1.44 (m, 2H), 1.44–1.26 (m, 2H). Hydrochloride salt: M.p. 210 °C. Elemental analyses (%) for C_17_H_22_N_2_O_3_·HCl Calc. C 60.26; N 8.27; H 6.84 found: C 60.37; N 8.11; H 6.97.

*2-(6-Morpholinohexyl)isoindoline-1,3-dione* (**6**). Procedure M1. Reaction of 2-(6-bromohexyl)isoindoline-1,3-dione [37] (0.65 g, 2.1 mmol) with morpholine (0.2 g, 2.3 mmol) and K_2_CO_3_ (0.87 g, 6.28 mmol) in acetonitrile (25 mL), after 20 h, column chromatography gave oil product. Yield 0.58 g (88%). TLC (S_3_) *R_f_* = 0.34. MW 316.18. Formula: C_18_H_24_N_2_O_3_. MS: *m*/*z* 317.26 [M + H]^+^. ^1^H-NMR (300 MHz, CDCl_3_) δ ppm: 7.86–7.80 (m, 2H), 7.73–7.66 (m, 2H), 3.72 (t, *J* = 4.5 Hz, 4H), 3.67 (t, *J* = 7.3 Hz, 2H), 2.45 (t, *J* = 4.7 Hz, 4H), 2.34 (t, *J* = 7.1 Hz, 2H), 1.75–1.59 (m, 2H), 1.58–1.46 (m, 2H), 1.44–1.19 (m, 4H). Hydrochloride salt: M.p. 195 °C. Elemental analyses (%) for C_18_H_24_N_2_O_3_·HCl Calc. C 61.27; N 7.94; H 7.14 found: C 60.93; N 7.87; H 7.23.

*2-(7-Morpholinoheptyl)isoindoline-1,3-dione* (**7**). Procedure M1. Reaction of 2-(7-bromoheptyl)isoindoline-1,3-dione [37] (0.648 g, 2 mmol) with morpholine (0.19 g, 2.2 mmol) and K_2_CO_3_ (0.83 g, 6 mmol) in acetonitrile (25 mL), after 20 h, column chromatography gave oil product. Yield 0.58 g (88%). TLC (S_3_) *R_f_* = 0.58. MW 330.19. Formula: C_19_H_26_N_2_O_3_. MS: *m*/*z* 331.42 [M + H]^+^. ^1^H-NMR (300 MHz, CDCl_3_) δ ppm: 7.88–7.75 (m, 2H), 7.76–7.62 (m, 2H), 3.70 (t, *J* = 4.7 Hz, 4H), 3.65 (t, *J* = 7.1 Hz, 2H), 2.41 (t, *J* = 4.6 Hz, 4H), 2.30 (t, *J* = 7.4 Hz, 2H), 1.77–1.56 (m, 2H), 1.53–1.20 (m, 8H). Hydrochloride salt: M.p. 142.5 °C. Elemental analyses (%) for C_19_H_26_N_2_O_3_·HCl Calc. C 62.20; N 7.64; H 7.42, found: C 62.11; N 8.06; H 7.48.

*2-(8-Morpholinooctyl)isoindoline-1,3-dione* (**8**) [57]. Procedure M1. Reaction of 2-(8-bromooctyl)isoindoline-1,3-dione [37] (0.676 g, 2 mmol) with morpholine (0.19 g, 2.2 mmol) and K_2_CO_3_ (0.83 g, 6 mmol) in acetonitrile (25 mL), after 20 h, column chromatography gave oil product. Yield 0.59 g (85%). TLC (S_3_) *R_f_* = 0.46. MW 344.21. Formula: C_20_H_28_N_2_O_3_. MS: *m*/*z* 345.38 [M + H]^+^. ^1^H-NMR (300 MHz, CDCl_3_) δ ppm: 7.86–7.78 (m, 2H), 7.74–7.63 (m, 2H), 3.75–3.68 (m, 4H), 3.65 (t, *J* = 7.2 Hz, 2H), 2.48–2.39 (m, 4H), 2.30 (t, *J* = 7.3 Hz, 2H), 1.71–1.59 (m, 2H), 1.51–1.42 (m, 2H), 1.35–1.24 (m, 8H). Hydrochloride salt: M.p. 133 °C. Elemental analyses (%) for C_20_H_28_N_2_O_3_·HCl Calc. C 63.06; N 7.35; H 7.67, found: C 63.21; N 7.38; H 7.75.

#### 3.1.4. General Procedure for the Synthesis of Compounds (**9**–**15**)

Procedure M2. A mixture of the appropriate 2-(bromoalkyl)-isoindoline-1,3-dione (1 equiv) with 1,2,3,4-tetrahydoisoquinoline (1 equiv) in the presence of K_2_CO_3_ (2.5-3 equiv) was stirred in acetonitrile under reflux for 20 h. Subsequently, the solvent was evaporated under vacuum, producing a residue which was further dissolved in 40 mL of sodium bicarbonate and extracted with ethyl acetate (3 × 30 mL). The organic layer was acidified 2 M·HCl and extracted with distilled water (3 × 30 mL). Then, the combined aqueous extracts were alkalized using 4M·NaOH, extracted with DCM and dried with anhydrous Na_2_SO_4_. The solvent was then evaporated and the residue was purified by silica gel column chromatography (S_4_) yielding a yellow oil. The final product was obtained in the form of hydrochloride salt. The following compounds were obtained.

*2-(7-(3,4-Dihydroisoquinolin-2(1H)-yl)heptyl)isoindoline-1,3-dione* (**12**). Procedure M2. Reaction of 2-(7-bromoheptyl)isoindoline-1,3-dione [37] (2 g, 6.2 mmol) with 1,2,3,4-tetrahydroisoquinoline (0.83 g, 6.2 mmol) and K_2_CO_3_ (2.57 g, 18.6 mmol) in acetonitrile (100 mL), after 20 h, column chromatography gave oil product. Yield 2.15 g (93%). TLC (S_4_) *R_f_* = 0.62. MW 376.22. Formula: C_24_H_28_N_2_O_2_. MS: *m*/*z* 377.29 [M + H]^+^. ^1^H-NMR (300 MHz, CDCl_3_) δ ppm: 7.89–7.77 (m, 2H), 7.76–7.63 (m, 2H), 7.15–6.94 (m, 4H), 3.72–3.56 (m, 4H), 2.89 (t, *J* = 5.9 Hz, 2H), 2.72 (t, *J* = 5.9 Hz, 2H), 2.53–2.41 (m, 2H), 1.76–1.50 (m, 4H), 1.43–1.28 (m, 6H). Hydrochloride salt: M.p. 167 °C. Elemental analyses (%) for C_24_H_28_N_2_O_2_·HCl Calc. C 69.8; N 6.78; H 7.08, found: C 70.10; N 6.83; H 7.47.

*2-(8-(3,4-Dihydroisoquinolin-2(1H)-yl)octyl)isoindoline-1,3-dione* (**13**). Procedure M2. Reaction of 2-(8-bromooctyl)isoindoline-1,3-dione [37] (2.1 g, 6.2 mmol) with 1,2,3,4-tetrahydroisoquinoline (0.83 g, 6.2 mmol) and K_2_CO_3_ (2.57 g, 18.6 mmol) in acetonitrile (100 mL), after 20 h, column chromatography gave oil product. Yield 2.33 g (96%). TLC (S_4_) *R_f_* = 0.64. MW 390.23. Formula: C_25_H_30_N_2_O_2_. MS: *m*/*z* 391.26 [M + H]^+^. ^1^H-NMR (300 MHz, CDCl_3_) δ ppm: 7.89–7.77 (m, 2H), 7.77–7.66 (m, 2H), 7.16–7.08 (m, 3H), 7.01–6.96 (m, 1H), 3.67 (t, *J* = 7.4 Hz, 2H), 3.61 (s, 2H), 2.90 (t, *J* = 5.9 Hz, 2H), 2.71 (t, *J* = 5.9 Hz, 2H), 2.48 (t, *J* = 7.5 Hz, 2H), 1.73–1.52 (m, 4H), 1.38–1.30 (m, 8H). Hydrochloride salt: M.p. 200 °C. Elemental analyses (%) for C_25_H_30_N_2_O_2_·HCl Calc. C 70.32; N 6.56; H 7.32, found: C 70.12; N 6.48; H 7.41.

*2-(10-(3,4-Dihydroisoquinolin-2(1H)-yl)decyl)isoindoline-1,3-dione* (**14**). Procedure M2. Reaction of 2-(10-bromodecyl)isoindoline-1,3-dione [51] (0.324 g, 1 mmol) with 1,2,3,4-tetrahydroisoquinoline (0.13 g, 1 mmol) and K_2_CO_3_ (0.41 g, 3 mmol) in acetonitrile (15 mL), after 20 h, column chromatography gave oil product. Yield 0.26 g (62%). TLC (S_4_) *R_f_* = 0.71. MW 418.26. Formula: C_27_H_34_N_2_O_2_. MS: *m*/*z* 419.43 [M + H]^+^. ^1^H-NMR (300 MHz, CDCl_3_) δ ppm: 7.90–7.77 (m, 2H), 7.76–7.63 (m, 2H), 7.16–6.96 (m, 4H), 3.67 (t, *J* = 7.2 Hz, 2H), 3.61 (s, 2H), 2.90 (t, *J* = 5.9 Hz, 2H), 2.72 (t, *J* = 5.9 Hz, 2H), 2.48 (t, *J* = 7.1 Hz, 2H), 1.73–1.50 (m, 4H), 1.38–1.24 (m, 12H). Hydrochloride salt: M.p. 140 °C. Elemental analyses (%) for C_27_H_34_N_2_O_2_·HCl Calc. C 71.27; N 6.16; H 7.75, found: C 7.35; N 6.18; H 7.83.

*2-(12-(3,4-Dihydroisoquinolin-2(1H)-yl)dodecyl)isoindoline-1,3-dione* (**15**) [60]. Procedure M2. Reaction of 2-(12-bromododecyl)isoindoline-1,3-dione [51] (0.31 g, 1 mmol) with 1,2,3,4-tetrahydroisoquinoline (0.13 g, 1 mmol) and K_2_CO_3_ (0.41 g, 3 mmol) in acetonitrile (15 mL), after 20 h, column chromatography gave oil product. Yield 0.25 g (57%). TLC (S_4_) *R_f_* = 0.73. MW 446.29. Formula: C_29_H_38_N_2_O_2_. MS: *m*/*z* 447.35 [M + H]^+^. ^1^H-NMR (300 MHz, CDCl_3_) δ ppm: 7.90–7.77 (m, 2H), 7.76–7.64 (m, 2H), 7.16–7.06 (m, 3H), 7.01–6.96 (m, 1H), 3.67 (t, *J* = 7.2 Hz, 2H), 3.62 (s, 2H), 2.90 (t, *J* = 5.9 Hz, 2H), 2.72 (t, *J* = 5.9 Hz, 2H), 2.49 (t, *J* = 7.5 Hz, 2H), 1.73–1.53 (m, 4H), 1.36–1.23 (m, 16H). Hydrochloride salt: M.p. 128 °C. Elemental analyses (%) for C_29_H_38_N_2_O_2_·HCl Calc. C 72.1; N 5.8; H 8.14, found: C 71.87; N 5.91; H 8.29.

#### 3.1.5. General Procedure for the Synthesis of Compounds (**20**–**23**)

Procedure M3. A mixture of an appropriate aminoalkyl-1,2,3,4-tetrahydroisoquinoline derivative (1 equiv) with dibromoethane (2 equiv) in the presence of K_2_CO_3_ (2 equiv) was stirred in acetonitrile at room temperature for 48 h. Subsequently, the solvent was evaporated under vacuum, producing a residue which was further dissolved in 20 mL of water and extracted with DCM (3 × 30 mL). The organic layer was dried with anhydrous Na_2_SO_4_. The solvent was then evaporated and the residue was purified by silica gel column chromatography (DCM, petroleum ether, MeOH, TEA; 5:3.5:1:3 drops). The final product was obtained in the form of hydrochloride salt. The following compounds were obtained.

*5-(3,4-dihydroisoquinolin-2(1H)-yl)-N,N-diethylpentan-1-amine* (**20**). Procedure M3. Reaction of 5-(3,4-dihydroisoquinolin-2(1*H*)-yl)pentan-1-amine [61] (**16**) (0.15 g, 0.69 mmol), bromoethane (0.15 g, 1.38 mmol) and K_2_CO_3_ (0.29 g, 2.07 mmol) in acetonitrile (30 mL), after 48 h, column chromatography gave oil product. Yield 0.16 g (84%). TLC (S_11_) *R_f_* = 0.54. MW 274.24. Formula: C_18_H_30_N_2_. MS: *m*/*z* 275.39 [M + H]^+^ 276.24. ^1^H-NMR (300 MHz, CDCl_3_) δ ppm: 7.16–7.04 (m, 3H), 7.01–6.96 (m, 1H), 3.61 (s, 2H), 2.90 (t, *J* = 6.1 Hz, 2H), 2.80–2.66 (m, 6H), 2.61 (t, *J* = 8.2 Hz, 2H), 2.50 (t, *J* = 7.5 Hz, 2H), 1.71–1.54 (m, 4H), 1.45–1.29 (m, 2H), 1.14 (t, *J* = 7.2 Hz, 6H). Hydrochloride salt: M.p. 245 °C. Elemental analyses (%) for C_18_H_30_N_2_·2HCl Calc. C 62.24; N 8.06; H 9.29, found: C 62.19; N 8.01; H 8.98.

*6-(3,4-Dihydroisoquinolin-2(1H)-yl)-N,N-diethylhexan-1-amin*e (**21**). Procedure M3. Reaction of 6-(3,4-dihydroisoquinolin-2(1*H*)-yl)hexan-1-amine (**17**) [53] (0.6 g, 2.58 mmol), bromoethane (0.57 g, 5.16 mmol) and K_2_CO_3_ (1.07 g, 7.74 mmol) in acetonitrile (30 mL), after 48 h, column chromatography gave oil product. Yield 0.55 g (74%). TLC (S_11_) *R_f_* = 0.38. *M*_W_ 288.26. Formula: C_19_H_32_N_2_. MS [M + H]^+^ 289.39. ^1^H-NMR (300 MHz, CDCl_3_) δ ppm: 7.14–7.03 (m, 3H), 6.98–6.94 (m, 1H), 3.60 (s, 2H), 2.89 (t, *J* = 5.9 Hz, 2H), 2.71 (t, *J* = 6.0 Hz, 2H), 2.61–2.35 (m, 6H), 1.65–1.39 (m, 4H), 1.37–1.26 (m, 6H), 1.02 (m, 6H). Hydrochloride salt: M.p. 215 °C. Elemental analyses (%) for C_19_H_32_N_2_·2HCl Calc. C 63.15; N 7.75; H 9.48, found: C 63.12; N 7.68; H 9.28.

*7-(3,4-Dihydroisoquinolin-2(1H)-yl)-N,N-diethylheptan-1-amine* (**22**). Procedure M3. Reaction of 7-(3,4-dihydroisoquinolin-2(1*H*)-yl)heptan-1-amine (**18**) [54] (0.16 g, 0.65 mmol), bromoethane (0.14 g, 1.3 mmol) and K_2_CO_3_ (0.27 g, 1.95 mmol) in acetonitrile (25 mL), after 48 h, column chromatography gave oil product. Yield 0.06 g (31%). TLC (S_6_) *R_f_* = 0.40. MW 302.27. Formula: C_20_H_34_N_2_. MS [M + H]^+^ 303.41. ^1^H-NMR (300 MHz, CDCl_3_) δ ppm: 7.12–7.07 (m, 3H), 7.04–6.98 (m, 1H), 3.61 (s, 2H), 2.89 (t, *J* = 5.9 Hz, 2H), 2.78–2.67 (m, 4H), 2.60 (t, *J* = 7.3 Hz, 2H), 2.48 (t, *J* = 7.3 Hz, 2H), 1.65–1.50 (m, 4H), 1.41–1.23 (m, 8H), 1.14 (t, *J* = 7.1 Hz, 6H). Hydrochloride salt: M.p. 197 °C. Elemental analyses (%) for C_20_H_34_N_2_·2HCl Calc. C 63.99; N 7.46; H 9.67, found: C 63.97; N 7.44; H 9.37.

*8-(3,4-Dihydroisoquinolin-2(1H)-yl)-N,N-diethyloctan-1-amine* (**23**). Procedure M3. Reaction of 8-(3,4-dihydroisoquinolin-2(1*H*)-yl)octan-1-amine (**19**) [54] (0.6 g, 2.31 mmol), bromoethane (0.503 g, 4.62 mmol) and K_2_CO_3_ (0.96 g, 6.93 mmol) in acetonitrile (30 mL), after 48 h, column chromatography gave oil product. Yield 0.45 g (62%). TLC (S_6_) *R_f_* = 0.44. MW 316.29. Formula: C_21_H_36_N_2_. MS [M + H]^+^ 317.39. ^1^H-NMR (300 MHz, CDCl_3_) δ ppm: 7.14–7.09 (m, 3H), 7.03–6.97 (m, 1H), 3.61 (s, 2H), 2.89 (t, *J* = 5.9 Hz, 2H), 2.71 (t, *J* = 5.7 Hz, 2H), 2.60–2.35 (m, 6H), 1.67–1.52 (m, 2H), 1.50–1.39 (m, 2H), 1.39–1.23 (m, 10H), 1.02 (t, *J* = 7.2 Hz, 6H). Hydrochloride salt: M.p. 183 °C. Elemental analyses (%) for C_21_H_36_N_2_·2HCl Calc. C 64.77; N 7.19; H 9.83, found: C 64.66; N 7.09; H 9.56.

#### 3.1.6. General Procedure for the Synthesis of Diethylamine Derivatives of *N*-alkyl-1*H*-indole (**30**–**35**)

Procedure M4. A mixture of the appropriate 1-(bromoalkyl)-1*H*-indole (1 equiv) with diethyl amine (4 equiv) in the presence of K_2_CO_3_ (1 equiv) was stirred in acetonitrile under reflux for 24 h. Subsequently, the solvent was evaporated under vacuum, producing a residue which was further dissolved in 40 mL of sodium bicarbonate and extracted with ethyl acetate (3 × 30 mL). The organic layer was dried with anhydrous Na_2_SO_4_. The solvent was then evaporated and the residue was purified by silica gel column chromatography (S_3_) yielding a yellow oil. The final product was obtained in the form of oxalic salt. The following compounds were obtained.

*N,N-diethyl-3-(1H-indol-1-yl)propan-1-amine* (**30**). Procedure M4. Reaction of 1-(3-bromopropyl)-1*H*-indole [60] (0.35 g, 1.47 mmol) with diethylamine (0.43 g, 5.8 mmol) and K_2_CO_3_ (0.2 g, 1.47 mmol) in acetonitrile (25 mL), after 24 h, column chromatography gave oil product. Yield 0.21 g (62%). TLC (S_1_) *R_f_* = 0.60. MW 230.18. Formula: C_15_H_22_N_2_. MS: *m*/*z* 231.31 [M + H]^+^. ^1^H-NMR (300 MHz, CDCl_3_) δ ppm: 7.63 (dd, *J* = 7.8, 0.8 Hz, 1H), 7.37 (dd, *J* = 8.2, 0.9 Hz, 1H), 7.24–7.16 (m, 1H), 7.15–7.01 (m, 2H), 6.49 (dd, *J* = 3.2, 0.9 Hz, 1H), 4.18 (t, *J* = 7.0 Hz, 2H), 2.51 (q, *J* = 7.2 Hz, 4H), 2.43 (t, *J* = 7.2, 6.8 Hz, 2H), 2.04–1.90 (m, 2H), 1.00 (t, *J* = 7.1 Hz, 6H). Oxalic acid salt: M.p. 156 °C. Elemental analyses (%) for C_15_H_22_N_2_·(COOH)_2_ Calc. C 63.73; N 8.74; H 7.55; found: C 63.89; N 8.82; H 7.63.

*N,N-diethyl-4-(1H-indol-1-yl)butan-1-amine* (**31**). Procedure M4. Reaction of 1-(4-bromobutyl)-1*H*-indole [53] (1.51 g, 6 mmol) with diethylamine (1.76 g, 24 mmol) and K_2_CO_3_ (0.83 g, 6 mmol) in acetonitrile (100 mL), after 24 h, column chromatography gave oil product. Yield 1.1 g (76%). TLC (S_1_) *R_f_* = 0.56. *M*_W_ 244.19. Formula: C_16_H_24_N_2_. MS: *m*/*z* 245.45 [M + H]^+^. ^1^H-NMR (300 MHz, CDCl_3_) δ ppm: 7.65 (d, *J* = 7.6 Hz, 1H), 7.37 (d, *J* = 8.2 Hz, 1H), 7.27–7.18 (m, 1H), 7.16–7.05 (m, 2H), 6.50 (d, *J* = 3.1 Hz, 1H), 4.15 (t, *J* = 7.1 Hz, 2H), 2.50 (q, *J* = 7.2 Hz, 4H), 2.42 (t, *J* = 7.8, 7.2 Hz, 2H), 1.95–1.78 (m, 2H), 1.56–1.38 (m, 2H), 1.00 (t, *J* = 7.2 Hz, 6H). Oxalic acid salt: M.p. 107 °C. Elemental analyses (%) for C_16_H_24_N_2_·(COOH)_2_ Calc. C 64.65; N 8.38; H 7.84, found: C 64.73; N 8.35; H 7.96.

*N,N-Diethyl-5-(1H-indol-1-yl)pentan-1-amine* (**32**). Procedure M4. Reaction of 1-(5-bromopentyl)-1*H*-indole [53] (1.33 g, 5 mmol) with diethylamine (1.5 g, 20 mmol) and K_2_CO_3_ (0.69 g, 5 mmol) in acetonitrile (80 mL), after 24 h, column chromatography gave oil product. Yield 0.89 g (69%). TLC (S_1_) *R_f_* = 0.61. *M*_W_ 258.21. Formula: C_17_H_26_N_2_. MS: *m*/*z* 259.47 [M + H]^+^. ^1^H-NMR (300 MHz, CDCl_3_) δ ppm: 7.58–7.43 (m, 2H), 7.37 (d, *J* = 3.1 Hz, 1H), 7.18–7.07 (m, 1H), 7.06–6.94 (m, 1H), 6.42 (d, *J* = 3.1 Hz, 1H), 4.18 (t, *J* = 6.9 Hz, 2H), 3.02 (q, *J* = 7.2 Hz, 4H), 2.90 (t, *J* = 8.7, 8.2 Hz, 2H), 1.87–1.71 (m, 2H), 1.71–1.53 (m, 2H), 1.37–1.19 (m, 2H), 1.09 (t, *J* = 7.0 Hz, 6H). Oxalic acid salt: M.p. 82 °C. Elemental analyses (%) for C_17_H_26_N_2_·(COOH)_2_ Calc. C 65.49; N 8.04; H 8.10, found: C 65.69; N 7.96; H 8.18.

*N,N-Diethyl-6-(1H-indol-1-yl)hexan-1-amine* (**33**). Procedure M4. Reaction of 1-(6-bromohexyl)-1*H*-indole [54] (0.8 g, 2.85 mmol) with diethylamine (0.83 g, 11.4 mmol) and K_2_CO_3_ (0.39 g, 2.85 mmol) in acetonitrile (45 mL), after 24 h, column chromatography gave oil product. Yield 0.59 g (76%). TLC (S_1_) *R_f_* = 0.68. MW 272.23. Formula: C_18_H_28_N_2_. MS: *m*/*z* 273.35 [M + H]^+^. ^1^H-NMR (300 MHz, CDCl_3_) δ ppm: 7.64 (d, *J* = 7.9 Hz, 1H), 7.35 (d, *J* = 8.2 Hz, 1H), 7.29–7.16 (m, 1H), 7.14–7.05 (m, 2H), 6.49 (d, *J* = 3.2 Hz, 1H), 4.12 (t, *J* = 7.1 Hz, 2H), 2.51 (q, *J* = 7.2 Hz, 4H), 2.38 (t, *J* = 8.0, 7.0 Hz, 2H), 1.51–1.21 (m, 8H), 1.02 (t, *J* = 7.2 Hz, 6H). Oxalic acid salt: M.p. 80 °C. Elemental analyses (%) for C_18_H_28_N_2_·(COOH)_2_ Calc. C 66.27; N 7.73; H 8.34, found: C 66.37; N 7.64; H 8.36.

*N,N-Diethyl-7-(1H-indol-1-yl)heptan-1-amine* (**34**). Procedure M4. Reaction of 1-(7-bromoheptyl)-1*H*-indole [54] (1.03 g, 3.5 mmol) with diethylamine (1.02 g, 14 mmol) and K_2_CO_3_ (0.48 g, 3.5 mmol) in acetonitrile (50 mL), after 24 h, column chromatography gave oil product. Yield 0.63 g (63%). TLC (S_1_) *R_f_* = 0.63. MW 286.24. Formula: C_19_H_30_N_2_. MS: *m*/*z* 287.35 [M + H]^+^. ^1^H-NMR (300 MHz, CDCl_3_) δ ppm: 7.69–7.59 (m, 1H), 7.42–7.31 (m, 1H), 7.25–7.17 (m, 1H), 7.14–7.07 (m, 2H), 6.49 (d, *J* = 3.1 Hz, 1H), 4.11 (t, *J* = 7.1 Hz, 2H), 2.52 (q, *J* = 7.2 Hz, 4H), 2.39 (t, *J* = 7.7 Hz, 2H), 1.92–1.75 (m, 2H), 1.43–1.23 (m, 8H), 1.02 (t, *J* = 7.2 Hz, 6H). Oxalic acid salt: M.p. 84 °C. Elemental analyses (%) for C_19_H_30_N_2_·(COOH)_2_ Calc. C 66.99; N 7.44; H 8.67, found: C 67.07; N 7.60; H 8.61.

*N,N-Diethyl-8-(1H-indol-1-yl)octan-1-amine* (**35**). Procedure M4. Reaction of 1-(8-bromooctyl)-1*H*-indole [54] (0.96 g, 3.1 mmol) with diethylamine (0.91 g, 12.4 mmol) and K_2_CO_3_ (0.43 g, 3.1 mmol) in acetonitrile (50 mL), after 24 h, column chromatography gave oil product. Yield 0.69 g (73%). TLC (S_1_) *R_f_* = 0.65. *M*_W_ 300.26. Formula: C_20_H_32_N_2_. MS: *m*/*z* 301.39 [M + H]^+^. ^1^H-NMR (300 MHz, CDCl_3_) δ ppm: 7.64 (d, *J* = 7.8 Hz, 1H), 7.40–7.31 (m, 1H), 7.25–7.16 (m, 1H), 7.14–7.06 (m, 2H), 6.49 (dd, *J* = 3.1, 0.8 Hz, 1H), 4.12 (t, *J* = 7.3 Hz, 2H), 2.52 (q, *J* = 7.2 Hz, 4H), 2.42–2.35 (m, 2H), 1.51–1.16 (m, 12H), 1.02 (t, *J* = 7.2 Hz, 6H). Oxalic acid salt: M.p. 87 °C. Elemental analyses (%) for C_20_H_32_N_2_·(COOH)_2_ Calc. C 67.66; N 7.17; H 8.78; found: C 67.47; N 7.05; H 8.71.

#### 3.1.7. General Procedure for the Synthesis of Compounds (**39**–**44**)

Procedure M5. To a solution of the appropriate 2-(bromoalkyl)benzo[*d*]isothiazol-3(2*H*)-one 1,1-dioxide (1 equiv) in DMSO was added threefold access of appropriate benzylamine (3 equiv) and the reaction mixture was heated to 60 °C for 3.5 h. Once the reaction was finished, 50 mL of water was added. The reaction mixture was extracted with dichloromethane (6 × 15 mL) and the combined organic extracts were washed with water (5 × 50 mL). Then, the organic phase was dried over anhydrous Na_2_SO_4_, filtered and concentrated under reduced pressure. Purification by flash chromatography (silica, dichloromethane to dichloromethane/methanol 94:6), afforded a yellow oil. The following compounds were obtained.

*2-(5-(2-fluorobenzylamino)pentyl)benzo[d]isothiazol-3(2H)-one 1,1-dioxide* (**39**). Procedure M5. Reaction of 2-(5-bromopentyl)benzo[*d*]isothiazol-3(2*H*)-one 1,1-dioxide [62] (**36**) (166 mg, 0.5 mmol) with 2-fluorobenzylamine (187 mg; 1.5 mmol) in DMSO (6.5 mL), after 3.5 h then purification by flash chromatography and column chromatography (CHCl_3_:MeOH:NH_3_—10:0.2:0.05) gave product **39**. Yield 38 mg (20%). TLC (S_3_) *R_f_* = 0.23. MW 376.45. Formula: C_19_H_21_FN_2_O_3_S. MS: *m*/*z* 377.08 [M + H]^+^. ^1^H-NMR (300 MHz, CDCl_3_) δ ppm: 8.08–8.02 (m, 1H), 7.94–7.78 (m, 3H), 7.33 (td, *J* = 7.57, 1.80 Hz, 1H), 7.26–7.16 (m, 1H), 7.13–6.96 (m, 2H), 3.83 (s, 2H), 3.81–3.73 (m, 2H), 2.63 (t, *J* = 7.05 Hz, 2H), 1.87 (dt, *J* = 15.13, 7.57 Hz, 2H), 1.64–1.52 (m, 3H), 1.52–1.39 (m, 2H). ^13^C-NMR (75 MHz, CDCl_3_) δ ppm: 161.1 (d, *J*_C-F_ = 245.0 Hz), 158.9, 137.6, 134.6, 134.2, 130.3 (d, *J*_C-F_ = 4.4 Hz), 128.5 (d, *J*_C-F_ = 7.7 Hz), 127.3, 127.2 (d, *J*_C-F_ = 15.0 Hz), 125.0, 124.0 (d, *J*_C-F_ = 3.3 Hz), 120.8, 115.2 (d, *J*_C-F_ = 21.5 Hz), 48.9, 47.2, 39.3, 29.6, 29.4, 28.3, 28.7, 24.5.

*2-(6-(2-fluorobenzylamino)hexyl)benzo[d]isothiazol-3(2H)-one 1,1-dioxide* (**40**). Procedure M5. Reaction of 2-(6-bromohexyl)benzo[*d*]isothiazol-3(2*H*)-one 1,1-dioxide [62] (**37**) (173 mg, 0.5 mmol) with 2-fluorobenzylamine (187 mg; 1.5 mmol) in DMSO (6.5 mL), after 3.5 h and purification by flash chromatography gave product **40**. Yield 133 mg (68 %). TLC (S_3_) *R_f_* = 0.24. MW 390.47. Formula: C_20_H_23_FN_2_O_3_S. MS: *m*/*z* 391.11 [M + H]^+^. ^1^H-NMR (300 MHz, CDCl_3_) δ ppm: 8.13–7.99 (m, 1H), 7.98–7.71 (m, 3H), 7.34 (td, *J* = 7.57, 1.80 Hz, 1H), 7.25–7.18 (m, 1H), 7.14–6.98 (m, 2H), 3.84 (s, 2H), 3.77 (t, *J* = 7,44 Hz, 2H), 2.62 (t, *J* = 7.05 Hz, 2H), 1.85 (quin, *J* = 7.37 Hz, 2H), 1.65 (br. s., 1H), 1.59–1.48 (m, 2H), 1.46–1.31 (m, 4H). ^13^C-NMR (75 MHz, CDCl_3_) δ ppm 161.1 (d, *J*_C-F_ = 245.0 Hz), 158.9, 137.6, 134.6, 134.2, 130.4 (d, *J*_C-F_ = 4.4 Hz), 128.6 (d, *J*_C-F_ = 8.3 Hz), 127.4, 127.1 (d, *J*_C-F_ = 14.9 Hz), 125.1, 124.0 (d, *J*_C-F_ = 3.9 Hz), 120.8, 115.2 (d, *J*_C-F_ = 22.1 Hz), 49.0, 47.2, 39.3, 29.7, 28.3, 26.7, 26.6.

*2-(7-(2-fluorobenzylamino)heptyl)benzo[d]isothiazol-3(2H)-one 1,1-dioxide* (**41**). Procedure M5. Reaction of 2-(7-bromoheptyl)benzo[d]isothiazol-3(2*H*)-one 1,1-dioxide [62] (**38**) (180 mg, 0.5 mmol) with 2-fluorobenzylamine (187 mg; 1.5 mmol) in DMSO (6.5 mL), after 3.5 h then purification by flash chromatography and column chromatography (CH_2_Cl_2_:MeOH—9.5:0.5) gave product **41**. Yield 90 mg (45%). TLC (S_3_) *R_f_* = 0.19. MW 404.50. Formula: C_21_H_25_FN_2_O_3_S. MS: *m*/*z* 405.13 [M + H]^+^. ^1^H-NMR (300 MHz, CDCl_3_) δ ppm: 8.08–8.02 (m, 1H), 7.95–7.78 (m, 3H), 7.35 (td, *J* = 7.44, 1.80 Hz, 1H), 7.26–7.19 (m, 1H), 7.14–6.98 (m, 2H), 3.85 (s, 2H), 3.76 (t, *J* = 7,44 Hz, 2H), 2.62 (t, *J* = 7.18 Hz, 2H), 2.04 (br. s., 1H), 1.85 (quin, *J* = 7.44 Hz, 2H), 1.52 (quin, *J* = 6.99 Hz, 2H), 1.46–1.30 (m, 6H). ^13^C-NMR (75 MHz, CDCl_3_) δ ppm 161.2 (d, *J*_C-F_ = 245.5 Hz), 158.9, 137.7, 134.6, 134.2, 130.4 (d, *J*_C-F_ = 5.0 Hz), 128.6 (d, *J*_C-F_ = 8.3 Hz), 127.4, 126.9 (d, *J*_C-F_ = 15.5 Hz), 125.1, 124.0 (d, *J*_C-F_ = 3.3 Hz), 120.8, 115.2 (d, *J*_C-F_ = 21.5 Hz), 49.0, 47.1, 39.4, 29.7, 28.9, 28.3, 27.1, 26.7.

*2-(5-(3-chlorobenzylamino)pentyl)benzo[d]isothiazol-3(2H)-one 1,1-dioxide* (**42**). Procedure M5. Reaction of 2-(5-bromopentyl)benzo[*d*]isothiazol-3(2*H*)-one 1,1-dioxide [62] (**36**) (166 mg, 0.5 mmol) with 3-chlorobenzylamine (212 mg; 1.5 mmol) in DMSO (6.5 mL), after 3.5 h and purification by flash chromatography gave product **42**. Yield 98 mg (50%). TLC (S_3_) *R_f_* = 0.29. *M*_W_ 392.90. Formula: C_19_H_21_ClN_2_O_3_S. MS: *m*/*z* 393.10 [M + H]^+^. ^1^H-NMR (300 MHz, CDCl_3_) δ ppm 8.10–8.03 (m, 1H), 7.95–7.81 (m, 3H), 7.34–7.30 (m, 1H), 7.26–7.17 (m, 3H), 3.84–3.74 (m, 4H), 2.64 (t, *J* = 6.92 Hz, 2H), 1.88 (dt, *J* = 15.07, 7.47 Hz, 2H), 1.65–1.40 (m, 5H). ^13^C-NMR (75 MHz, CDCl_3_) δ ppm 158.93, 142.45, 137.60, 134.65, 134.27, 134.16, 129.58, 128.14, 127.36, 127.00, 126.17, 125.09, 120.86, 53.33, 48.98, 39.25, 29.38, 28.24, 24.43.

*2-(6-(3-chlorobenzylamino)hexyl)benzo[d]isothiazol-3(2H)-one 1,1-dioxide* (**43**). Procedure M5. Reaction of 2-(6-bromohexyl)benzo[*d*]isothiazol-3(2*H*)-one 1,1-dioxide [62] (**37**) (166 mg, 0.48 mmol) with 3-chlorobenzylamine (212 mg; 1.5 mmol) in DMSO (6.5 mL), after 3.5 h and purification by flash chromatography gave product **43**. Yield 112 mg (58%). TLC (S_3_) *R_f_* = 0.31. *M*_W_ 406.93. Formula: C_20_H_23_ClN_2_O_3_S. MS: *m*/*z* 407.06 [M + H]^+^. ^1^H-NMR (300 MHz, CDCl_3_) δ ppm 8.10–8.02 (m, 1H), 7.96–7.77 (m, 3H), 7.36–7.30 (m, 1H), 7.26–7.17 (m, 3H), 3.83–3.70 (m, 4H), 2.62 (t, *J* = 7.05 Hz, 2H), 1.86 (quin, *J* = 7.37 Hz, 2H), 1.62 (br. s., 1H), 1.59–1.47 (m, 2H), 1.47–1.38 (m, 4H). ^13^C-NMR (75 MHz, CDCl_3_) δ ppm 158.92, 142.42, 137.67, 134.61, 134.23, 134.18, 129.58, 128.17, 127.41, 127.02, 126.19, 125.08, 120.84, 53.33, 49.12, 39.31, 29.73, 28.30, 26.65, 26.59.

*2-(7-(3-chlorobenzylamino)heptyl)benzo[d]isothiazol-3(2H)-one 1,1-dioxide* (**44**). Procedure M5. Reaction of 2-(7-bromoheptyl)benzo[*d*]isothiazol-3(2*H*)-one 1,1-dioxide [62] (**38**) (180 mg, 0.5 mmol) with 3-chlorobenzylamine (212 mg; 183 μL, 1.5 mmol) in DMSO (6.5 mL), after 3.5 h and purification by flash chromatography gave product **44**. Yield 134 mg (64%). TLC (S_3_) *R_f_* = 0.18. MW 420.95. Formula: C_21_H_25_ClN_2_O_3_S. MS: *m*/*z* 421.08 [M + H]^+^. ^1^H-NMR (300 MHz, CDCl_3_) δ ppm: 8.09–8.02 (m, 1H), 7.95–7.78 (m, 3H), 7.37–7.30 (m, 1H), 7.26–7.17 (m, 3H), 3.82–3.71 (m, 4H), 2.61 (t, *J* = 7.05 Hz, 2H), 1.85 (quin, *J* = 7.37 Hz, 2H), 1.72 (br. s., 1H), 1.58–1.45 (m, 2H), 1.45–1.30 (m, 6H). ^13^C-NMR (75 MHz, CDCl_3_) δ ppm 158.90, 142.26, 137.66, 134.61, 134.23, 134.18, 129.59, 128.20, 127.41, 127.05, 126.23, 125.06, 120.83, 53.30, 49.18, 39.36, 29.72, 28.84, 28.29, 27.03, 26.64.

### 3.2. Molecular Modelling

The three-dimensional ligand structures were built with Corina online tool. Subsequently, atom types and protonation states were checked and Gasteiger-Marsili charges were assigned using Sybyl 8.0. Finally, ligand structures were saved in the mol2 format. Docking to *Torpedo californica* AChE (PDB code: 1EVE) was performed using the Gold 4.1. The target was prepared as follows: all histidine residues were protonated at Nε, hydrogen atoms added, ligand molecules removed, and binding sites defined as all amino acid residues within 10 Å from donepezil. The presence of conserved water molecules was also taken into account. A standard set of genetic algorithms with a population size of 100, number of operations being 100,000 and with a clustering tolerance of 1 Å were applied. As a result, 10 ligand conformations were obtained and sorted according to ChemScore function values. Results were visualized using PyMOL.

### 3.3. Biological Evaluation

#### 3.3.1. *In vitro* Inhibition of AChE and BuChE

To assess the inhibitory activity of the target compounds towards cholinesterases, we followed Ellman’s assay (as modified for 24-well microplates) using AChE from *Electrophorus electricus* (*Ee*AChE) (Sigma–Aldrich) and BuChE from equine serum (*Eq*BuChE, Sigma–Aldrich). 500 U of AChE or BuChE was dissolved in 1 ml of a gelatine solution (1% in water) and diluted with demineralized water to give a stock solution of 5 U/mL. The AChE solution was further diluted before use to give a final concentration of 3.125 U/mL. The 0.0125 M 5,5′-dithiobis-(2-nitrobenzoic acid) (DTNB, Ellman’s reagent) solution containing 0.15% (*w*/*v*) sodium carbonate and the 0.01875 M acetylthiocholine (ATC) iodide solution were prepared in demineralized water. All assays were performed in 0.1 M phosphate buffer pH 8.0. The tested compounds or water in a blank sample (25 µL) were incubated with the enzyme (20 µL) for 5 min at 25 °C in buffer (765 µL) prior to starting the reaction. Then, DTNB (20 µL) and ATC (20 µL) were added. After 5 min of the reaction, changes in absorbance were measured at 412 nm using a microplate reader (EnSpire Multimode, PerkinElmer). Each condition was measured in triplicate. The percentages of enzyme inhibition were calculated from the equation 100%—A_i_/A_0_ × 100%, where A_i_ is the absorbance of a sample with an inhibitor and A_0_ is the absorbance of a blank sample (100% activity of the enzyme). The IC_50_ values were calculated using GraphPad Prism 5. Data is expressed as mean ± SEM.

#### 3.3.2. Kinetic Characterization of *Ee*AChE Inhibition

To estimate the type of inhibition of *Ee*AChE, the same experimental protocol as reported above (4.3.1) was performed. Different concentrations of the substrate ATC (0.067–0.5 mM) were used to create Lineweaver-Burk plots by plotting the inverse initial velocity (1/*V*) as a function of the inverse of the substrate concentration (1/[S]). The stock solution of ATC (0.5 mM in a well) was prepared in water and diluted before use to obtained 0.4, 0.3, 0.2, 0.1and 0.067 mM concentrations of substrate. The double reciprocal plots were assessed by a weighted least square analysis that assumed the variance of *V* to be a constant percentage of *V* for the entire data set. Each experiment was performed in triplicate. Then, to confirm the mode of inhibition, Cornish-Bowden plots were obtained by plotting S/*V* (substrate concentration/velocity ratio) *versus* the inhibitor concentration [I]. Data analysis was performed with GraphPad Prism 5.

#### 3.3.3. *In vitro* Inhibition of Aβ_1–42_ Aggregation

Thioflavin-T (ThT) fluorometric assay was performed to investigate the effect of the test compounds on the self-aggregation of Aβ_1–42_. Recombinant human HFIP-pretreated Aβ_1–42_ peptide (Lot number 2387442, Merck Millipore, Darmstadt, Germany) was dissolved in DMSO. Prior to the incubations, the Aβ_1–42_ peptide stock solution was diluted in 150 mM HEPES buffer (pH 7.4) containing 150 mM NaCl, to give a concentration of 7.5 μM. Then Aβ_1–42_ (20 μL) was mixed with of the test compounds (10 μL, 100 μM stock in HEPES; 10 μM final concentration), added to the corresponding wells in black-walled 96-well plates, and diluted with ThT solution (70 μL, 14.3 μM stock solution in HEPES; 10 μM final concentration), to the final volume of 100 μL (1.5 μM final Aβ_1–42_ concentration). Each sample was prepared in quadruplicate, and the DMSO was always at 3%. To quantify amyloid fibril formation, the ThT fluorescence was measured through the bottom of the plate every 180 s at an excitation wavelength of 440 nm and emission wavelength of 490 nm, with the medium continuously shaking between measurements using a 96-well microplate reader (Synergy™ H4, BioTek Instruments, Inc., Winooski, VT, USA). The ThT emission of the Aβ_1–42_ began to rise after 4 h, reached a plateau after 36 h, and remained almost unchanged for an additional 12 h of incubation. The fluorescence intensities at the plateau in the absence and presence of the test compounds were averaged, and the average fluorescence of the corresponding wells at t = 0 h was subtracted. The Aβ_1–42_ self-induced aggregation inhibitory potencies are expressed as the percentage inhibition (%inh = (1 − F_i_/F_0_) ×100%), where F_i_ is the increase in fluorescence of Aβ_1–42_ treated with the test compounds, and F_0_ is the increase in fluorescence of Aβ_1–42_ alone.

#### 3.3.4. PAMPA-BBB Assay

The brain penetration of compounds **4**, **7**, **13**, **39** and **42** was assessed using the parallel artificial membrane permeability assay for blood brain barrier (BBB-PAMPA). The BBB-PAMPA Explorer Test System was purchased from pION Inc. The *in vitro* permeability through BBB-1 lipid membrane was determined for 7 commercial drugs and the tested compounds. The compounds were dissolved in DMSO (10 mM stock solution) and diluted with Prisma HT buffer (5 µL/1 mL). Then, the acceptor 96-well microplate was filled with solution of the tested compounds in buffer (200 µL/well). The filter membrane in acceptor 96-well microplate was impregnated with BBB-1 lipid solution (5 µL/well) and the acceptor plate was filled with Brain Sink Buffer (200 µL/well). The acceptor plate was carefully placed on the donor plate to form a sandwich that was left undisturbed for 2 h at 37 °C. After incubation, the donor plate was carefully removed. The concentration of compounds in the acceptor, the donor, and the reference wells were measured using EnSpire Multimode Microplate Reader (PerkinElmer). Logarithm of the effective permeability (log P_e_) of the compounds was calculated using the pION software. Assay validation was done by comparison of the experimental permeability of the seven commercial drugs with their reference values established for this assay by pION. We established the following ranges of permeability: CNS+, log P_e_ > −4.5, high permeability (*i.e.*, can enter the CNS); CNS–, log P_e_ ≤−6.3, low permeability (*i.e.*, excluded from the CNS); CNS±, –6.3 < log P_e_ ≤−4.5, uncertain BBB permeability.

## 4. Conclusions

Herein, we presented a continuation of our studies focused on the search for multitarget compounds as potential anti-AD agents. With the aid of molecular modelling, we designed new dual binding site inhibitors of AChE. The designed compounds were synthesized and evaluated *in vitro*. We found that most of the target compounds are moderate or potent AChE inhibitors or dual AChE/BuChE inhibitors with IC_50_ values in the low micromolar and submicromolar range. Structure–activity relationship analysis revealed that among the tested compounds containing different heteroaromatic moieties, the most potent inhibitors were derivatives with a saccharin fragment with the most potent compound **42** (*Ee*AChE IC_50_ = 70 nM). Regarding the amine part of the molecules, we found that pyrrolidine, benzylamine and its rigid analogue—tetrahydroisoquinoline—are beneficial for AChE inhibitory potency when compared to morpholine. Compound **13**, a tetrahydroisoquinoline derivative, was found to be a dual inhibitor of AChE and BuChE (*Ee*AChE IC_50_ = 0.76 μM, *Eq*BuChE IC_50_ = 0.618 μM). This balanced potency seems to be a promising starting point for further studies given the fact that currently used anti-AD drugs inhibit these two enzymes with inhibitory potencies in the same order of magnitude (*i.e.*, rivastigmine: *Ee*AChE IC_50_ = 3.01–3.4 μM, *Eq*BuChE IC_50_ = 0.30–5.5 μM; galantamine: *Ee*AChE IC_50_ = 0.665–2.41μM, *Eq*BuChE IC_50_ = 17.38–19.78 μM) [63,64,65]. Compound **13** was also the most potent inhibitor of self-induced Aβ_1–42_ aggregation (35.80% at 10 μM) comparable with other multifunctional agents described recently [22] and a more potent inhibitor than donepezil (11.48% at 10 μM). Kinetic studies revealed that the developed derivatives are mixed or non-competitive AChE inhibitors. Molecular modelling studies indicated that all the compounds were dual binding site inhibitors able to interact with catalytic and peripheral active sites of AChE. The results of both kinetic studies and molecular modelling showed that the tested compounds display a similar mechanism of action to that of donepezil. The results from the PAMPA-BBB assay indicated that the tested compounds are able to cross the BBB *in vitro*. In conclusion, our studies have provided a better understanding of the structure–activity relationships in a group of heterodimeric cholinesterase inhibitors and allowed the identification of compounds **42** and **13** as interesting agents which can be used in the further development of potential anti-Alzheimer’s drugs.

## Figures and Tables

**Table 1:** Inhibitory potency of the synthesized compounds against *Ee*AChE, *Eq*BuChE and against Aβ_1–42_ aggregation. 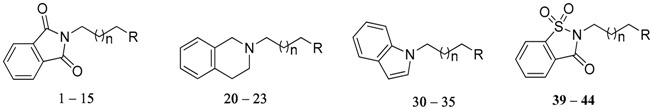

Compd	*R*	*n*	*Ee*AChE IC_50_ [µM] ^a^	*Eq*BuChE IC_50_ [µM] ^a^	Aβ_1–42_ Aggreg. ^b^
**1**	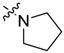	3	0.781 ± 0.009	>50 ^c^	-
**2**		4	0.643 ± 0.006	>50 ^c^	-
**3**		5	0.276 ± 0.003	>50 ^c^	-
**4**		6	0.282 ± 0.011	3.241 ± 0.141	15.17 ± 3.06
**5**	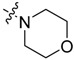	3	18.117 ± 0.402	>50 ^c^	-
**6**		4	17.249 ± 0.474	>50 ^c^	-
**7**		5	4.114 ± 0.066	>50 ^c^	17.06 ± 6.13
**8**		6	4.503 ± 0.064	>50 ^c^	-
**9**	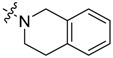	2	5.596 ± 0.087	12.818 ± 0.107	-
**10**		3	1.266 ± 0.011	5.314 ± 0.160	-
**11**		4	0.387 ± 0.003	5.124 ± 0.090	-
**12**		5	0.688 ± 0.017	1.169 ± 0.027	-
**13**		6	0.760 ± 0.017	0.618 ± 0.009	35.80 ± 5.39
**14**		8	>50 ^c^	1.727 ± 0.038	-
**15**		10	>50 ^c^	3.662 ± 0.085	-
**20**	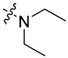	3	>50 ^c^	>50 ^c^	-
**21**		4	>50 ^c^	>50 ^c^	-
**22**		5	17.303 ± 0.401	37.129 ± 0.878	-
**23**		6	4.722 ± 0.149	14.002 ± 0.343	<10 ^d^
**30**	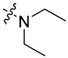	1	>50 ^c^	>50 ^c^	-
**31**		2	>50 ^c^	25.995 ± 0.813	-
**32**		3	>50 ^c^	22.571 ± 0.602	-
**33**		4	>50 ^c^	28.733 ± 0.723	-
**34**		5	22.185 ± 0.424	1.472 ± 0.593	-
**35**		6	9.557 ± 0.289	3.471 ± 0.100	16.64 ± 2.74
**39**	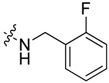	3	0.150 ± 0.003	>50 ^c^	12.27 ± 2.63
**40**		4	1.145 ± 0.015	>50 ^c^	-
**41**		5	0.735 ± 0.009	4.971 ± 0.127	-
**42**	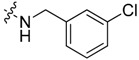	3	0.070 ± 0.001	>50 ^c^	16.54 ± 3.01
**43**		4	0.596 ± 0.006	6.535 ± 0.163	-
**44**		5	0.923 ± 0.012	2.459 ± 0.032	-
**Compd. I ^e^**			0.900 ± 0.004	>50 ^c^	30.10 ± 5.78 ^f^
**Compd. II ^e^**			0.036 ± 0.001	>50 ^c^	22.19 ± 16.6
**Donepezil**			0.010 ± 0.001	1.830 ± 0.176	11.48 ± 4.51
**Tacrine**			0.024 ± 0.001	0.002 ± 0.001	-

^a^ IC_50_ values are expressed as mean ± standard error of the mean (SEM) of at least three experiments; ^b^ % of inhibition at 10 µM compound concentration and 1.5 µM Aβ_1–42_; values are expressed as mean ± standard deviation (SD); ^c^ % Inhibition at 50 μM lower than 50%; ^d^ % Inhibition at 10 µM lower than 10%, compound defined as not active; *^e^* Data from ref. [37] for compound **I** and from [40] for compound **II**, respectively; ^f^ % of inhibition at 50 µM compound concentration, data for Aβ_12–23_, values are expressed as mean ± standard deviation (SD).

**Table 2:** Permeability (log P_e_) in the PAMPA-BBB assay for commercial drugs and the selected compounds with prediction of their BBB penetration.

Compound	Log P_e_^a^	Prediction
Verapamil	–3.9	CNS+
Lidocaine	–4.3	CNS+
Quinidine	–4.0	CNS+
Progesterone	–3.8	CNS+
Propranolol	–3.7	CNS+
Corticosterone	–4.5	CNS±
Theophylline	–6.3	CNS–
**4**	–4.0	CNS+
**7**	–4.2	CNS+
**13**	–4.2	CNS+
**39**	–4.0	CNS+
**42**	–4.3	CNS+

^a^ Data are means of two replicates (*n* = 2); CNS+, log P_e_ > −4.5, high permeability (*i.e.*, can enter the CNS); CNS–, log P_e_ ≤ −6.3, low permeability (*i.e*., excluded from the CNS); CNS±, −6.3 < log P_e_ ≤ −4.5, uncertain permeability

## Supplementary Materials

Supplementary materials can be accessed at: http://www.mdpi.com/1420-3049/21/4/410/s1.

## Abbreviations

The following abbreviations are used in this manuscript:

Ach	acetylcholine
AChE	acetylcholinesterase
AD	Alzheimer’s disease
Aβ	amyloid beta peptide
BBB	blood-brain barrier
BuChE	butyrylcholinesterase
BuChE	butyrylcholinesterase
CAS	catalytic anionic site in acetylcholinesterase
CNS	central nervous system
*Ee*AChE	acetylcholinesterase from electric eel
*Eq*BuChE	equine serum butyrylcholinesterase
MTDL	multi-target-directed ligands
NFTs	neurofibrillary tangles
PAMPA	parallel artificial membrane permeation assay
PAS	peripheral anionic site in acetylcholinesterase
*Tc*AChE	acetylcholinesterase from *Torpedo californica*
