# Supplementary material for: Synthesis, Molecular Modelling and Biological Evaluation of Novel Heterodimeric, Multiple Ligands Targeting Cholinesterases and Amyloid Beta
Source: Molecules. 2016 Mar 26;21(4):410. doi: 10.3390/molecules21040410 (PMC6273065; doi:10.3390/molecules21040410)
Supplement: Supplementary file 1 [file molecules-21-00410-s001.pdf]

Supplementary Materials: Synthesis, Molecular Modelling and Biological Evaluation of Novel Heterodimeric, Multiple Ligands Targeting Cholinesterases and Amyloid Beta

Michalina Hebda Marek Bajda, Anna Więckowska, Natalia Szałaj, Anna Pasieka, Dawid Panek, Justyna Godyń, Tomasz Wichur, Damijan Knez, Stanislav Gobec and Barbara Malawska

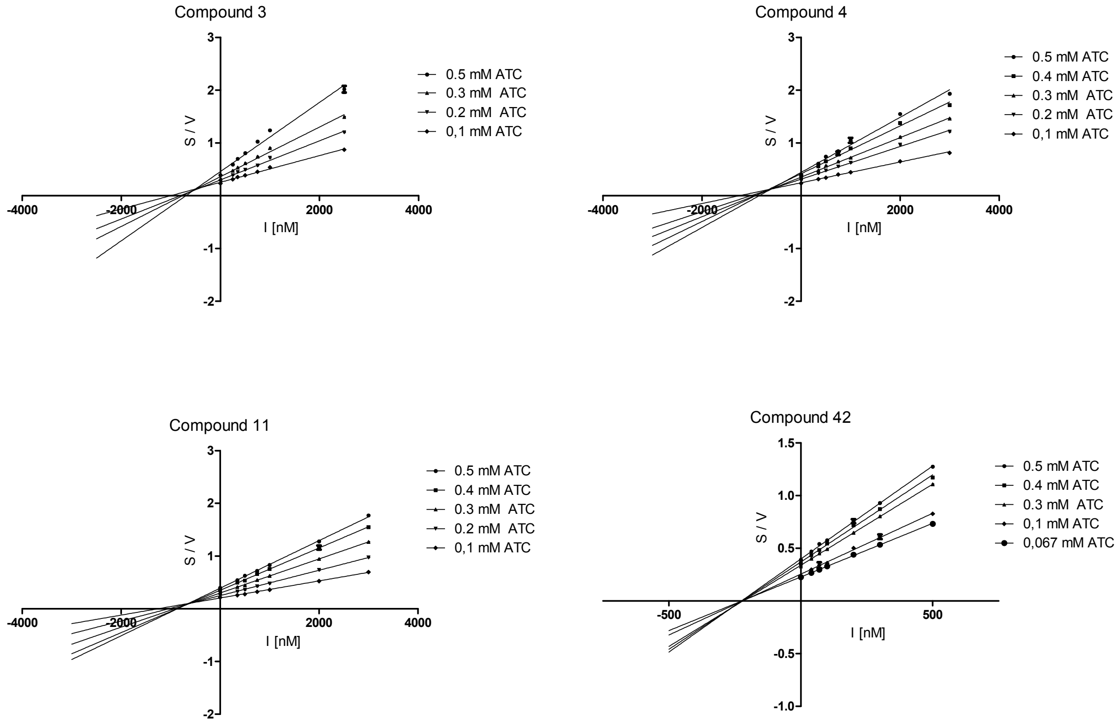

Figure S1. Cornish—Bowden plots illustrating mixed type of *Ee*AChE inhibition by compounds **3, 4, 11** and non-competitive type of *Ee*AChE inhibition by compound **42**. I = inhibitor concentration; S = substrate concentration, *V* = initial velocity rate.
